# An Experimental Design Approach for Impurity Profiling of Valacyclovir-Related Products by RP-HPLC

**DOI:** 10.3797/scipharm.1403-20

**Published:** 2014-05-05

**Authors:** Prakash Katakam, Baishakhi Dey, Nagiat T Hwisa, Fathi H Assaleh, Babu R Chandu, Rajeev K Singla, Analava Mitra

**Affiliations:** ^1^ Faculty of Pharmacy, University of Zawia, Az Zawiyah, Libya.; ^2^ School of Medical Science & Technology, IIT Kharaghpur, India.; ^3^ Division of Biotechnology, Netaji Subhas Institute of Technology, New Delhi, India.

**Keywords:** Impurity profiling, Box-Behnken design, Retention time, Validated method, Response Surface Methodology, Valaciclovir

## Abstract

Impurity profiling has become an important phase of pharmaceutical research where both spectroscopic and chromatographic methods find applications. The analytical methodology needs to be very sensitive, specific, and precise which will separate and determine the impurity of interest at the 0.1% level. Current research reports a validated RP-HPLC method to detect and separate valacyclovir-related impurities (Imp-E and Imp-G) using the Box-Behnken design approach of response surface methodology. A gradient mobile phase (buffer: acetonitrile as mobile phase A and acetonitrile: methanol as mobile phase B) was used. Linearity was found in the concentration range of 50–150 μg/mL. The mean recovery of impurities was 99.9% and 103.2%, respectively. The %RSD for the peak areas of Imp-E and Imp-G were 0.9 and 0.1, respectively. No blank interferences at the retention times of the impurities suggest the specificity of the method. The LOD values were 0.0024 μg/mL for Imp-E and 0.04 μg/mL for Imp-G and the LOQ values were obtained as 0.0082 μg/mL and 0.136 μg/mL, respectively, for the impurities. The S/N ratios in both cases were within the specification limits. Proper peak shapes and satisfactory resolution with good retention times suggested the suitability of the method for impurity profiling of valacyclovir-related drug substances.

## Introduction

Impurity profiling is a broad term which encompasses the identification, quantitative determination, and structural elucidation of impurities with the aid of spectroscopy or chromatographic techniques or the utilizations of the latest developed hyphenated methods [1, 2]. Quality is an essential attribute in any pharmaceutical product, which is greatly determined by the content of the active ingredient present in it whatever its source or origin and the techniques adopted for its synthesis or formulation. In the pharmaceutical world, impurities are considered as any material other than the active pharmaceutical ingredient (API) or excipients; may it be of organic or inorganic origin, may it arise from varied sources like process-related drug substances (starting material, intermediate, or drug product), an impurity in starting materials, degradation of drug substances, unwanted excipient-interactions, contaminations of some reagents and catalysts, presence of enantiomeric impurities, and some impurities may be due to environmental factors [1–6]. The presence of these unwanted chemicals, even in small amounts, may influence the efficacy and safety of the pharmaceutical products and can precipitate adverse and toxic drug reactions in patients after consumption. Thus, along with the purity profile, impurity profiling is now gaining critical attention from regulatory authorities. Various pharmacopoeias are slowly incorporating limits of allowable levels of impurities present in the APIs or formulations [3–5].

With the tremendous advancements of analytical technologies and changed perspectives of the research scenario, not only detection of active constituents, but a detailed profiling of impurities offers a broad scope of research in pharmaceutical and other bio-allied fields. The International Conference on Harmonization of Technical Requirements for Registration of Pharmaceuticals for Human Use (ICH) has also published guidelines for the validation of methods for analysing impurities in new drug substances and drug products, residual solvents, and microbiological impurities [1, 2]. ICH defines an impurity as “any chemical compound of the medicinal product which is not the chemical entity defined as the active substance or as an excipient in the product.” According to its guidelines, the threshold limit for any impurity is at 0.1% for drugs used in dosages more than 2 g/day and for those dosed at less than 2 g/day, the limit is below 0.05% [2, 6, 6]. If the structure and physical-chemical data-related information are available about the impurities, several spectroscopic and microchemical techniques have been developed which require minute quantities of material and readily enable the structural elucidation of the impurities. General techniques like TLC, HPLC, HPTLC, and AAS; and hyphenated techniques like LC–MS/MS, LC–NMR, LC–NMR–MS, NME–MS, GC–MS, and LC–MS find applications in impurity profiling. However, routine quality control demands very selective, yet simple and cost-effective analytical methods for monitoring impurities at the 0.1% level [1–6].

**Fig. 1.**
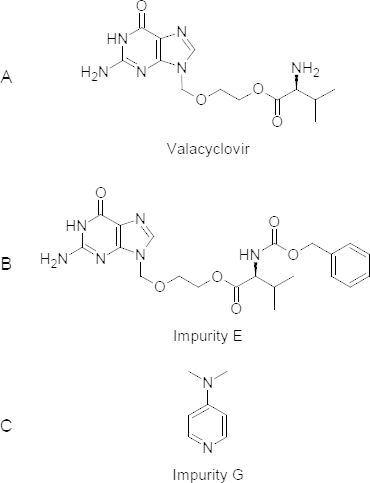
Chemical structures of (A) valacyclovir, (B) impurity E, and (C) impurity G

Valacyclovir (VCR) is a hydrochloride salt of the L-valyl ester of acyclovir. Chemically, it is 2-[(2-amino-6-oxo-1,6-dihydro-9*H*-purin-9-yl)methoxy]ethyl L-valinate (Figure 1A), used for the treatment of herpes simplex and herpes zoster. It is phosphorylated by viral thymidine kinase to acyclovir triphosphate (the active metabolite) which then inhibits herpes viral DNA replication by competitive inhibition of viral DNA polymerase, and by incorporation into and termination of the growing viral DNA chain [8–11].

The active ingredient VCR has two process-related impurities, one of which is 2-[(2-amino-6-oxo-1,6-dihydro-9*H*-purin-9-yl)methoxy]ethyl *N*-[(benzyloxy)carbonyl]-L-valinate, with the molecular formula C_21_H_26_N_6_O_6_ (Figure 1B), formed as an intermediate in the first stage of the synthesis of VCR and it is designated as impurity E. Another one is 4-(dimethyl-amino)pyridine (DMAP), a derivative of pyridine with the chemical formula (CH_3_)_2_NC_5_H_4_N (Figure 1C) and used as a catalyst in the synthesis of VCR, is designated as impurity G. Impurity G has a relatively high toxicity, is corrosive to the lungs and eyes, and gets absorbed through the skin [8]. Both impurities E and G are official in the US and European pharmacopeias.

Factorial experiments find extensive applications in all research fields which allow the investigation of multiple factors simultaneously and also the examination of one factor at different levels of the other factor or factors. The Box-Behnken experimental design (BBD), developed by Box and Behnken in 1980 is a useful response surface methodology, where the level of one of the factors is fixed at the centre level while combinations of all the levels of the other factors are applied [8–10]. Extensive literature surveys have reported several analytical techniques for the estimation of VCR in both bulk and dosage forms [11–23]. To the best of our knowledge, the application of the experimental design approach was not found in the development of analytical methods of HPLC for VCR. Therefore, the current research aims to devise an RP–HPLC method for impurity profiling of VCR-related impurities E and G at the specification limit for its effective application as a quality control tool are the process technology and validation parameters optimized by the BBD approach under response surface methodology (RSM). Here lies the novelty of our approach in the development of the analytical method for impurity profiling of VCR.

**Tab. 1.** Design matrix of BBD model for optimizing chromatographic conditions

Std. order	Run order	Pt Type	Blocks	Buffer in mobile phase A (%)	ACN in mobile phase B (%)	Flow rate (mL/min)	Column temp. (°C)	Retention time (min)	RT ratio	Peak area (%)
1	2	1	90	90	0.9	27.5	6.7	0.41	28.1
13	2	2	1	95	90	0.8	27.5	7.4	0.47	30.1
26	3	0	1	95	95	0.9	27.5	6.7	0.41	28.1
23	4	2	1	95	90	0.9	30	6.7	0.41	28.1
17	5	2	1	90	95	0.8	27.5	7.1	0.6	8.61
22	6	2	1	95	100	0.9	25	6.7	0.41	28.1
16	7	2	1	95	100	1	27.5	4.9	0.89	18.7
11	8	2	1	90	95	0.9	30	6.7	0.41	28.1
15	9	2	1	95	90	1	27.5	9.2	0.53	32.8
7	10	2	1	95	95	0.8	30	7.2	0.59	0.16
20	11	2	1	100	95	1	27.5	4.9	0.89	18.7
24	12	2	1	95	100	0.9	30	6.9	0.43	29.1
9	13	2	1	90	95	0.9	25	6.9	0.41	28.3
2	14	2	1	100	90	0.9	27.5	6.9	0.43	28.1
8	15	2	1	95	95	1	30	9.2	32.8	0.53
21	16	2	1	95	90	0.9	25	6.7	0.41	29.3
4	17	2	1	100	100	0.9	27.5	6.7	0.4	28.1
14	18	2	1	95	100	0.8	27.5	7.4	0.48	30.1
27	19	0	1	95	95	0.9	27.5	6.7	0.41	29.7
5	20	2	1	95	95	0.8	25	7.4	0.47	8.57
18	21	2	1	100	95	0.8	27.5	7.4	0.47	8.61
6	22	2	1	95	95	1	25	9.2	0.53	32.8
12	23	2	1	100	95	0.9	30	6.7	0.41	28.7
25	24	0	1	95	95	0.9	27.5	6.7	0.43	28.1
3	25	2	1	90	100	0.9	27.5	6.7	0.41	29.1
19	26	2	1	90	95	1	27.5	4.9	0.89	18.7
10	27	2	1	100	95	0.9	25	6.7	0.41	28.3

## Results and Discussion

**Fig. 2.**
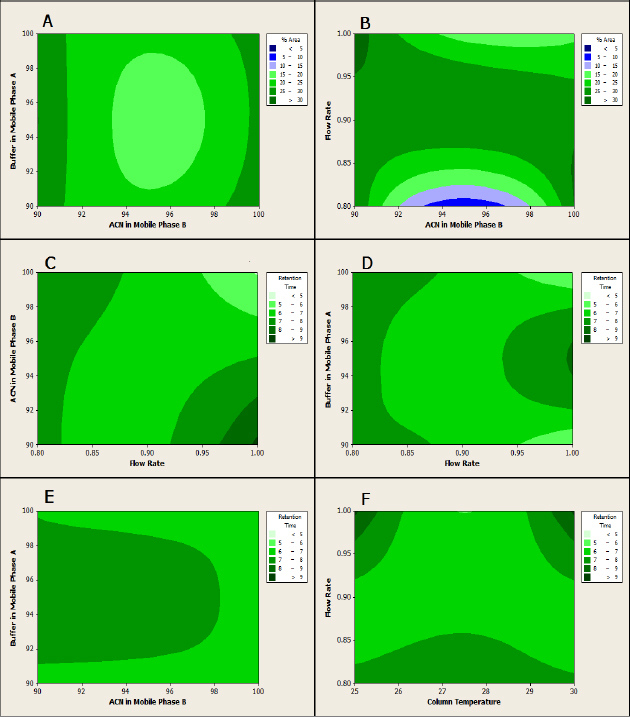
Contour plots of (A) percentage area vs. buffer and ACN concentrations in mobile phase A, (B) percentage area vs. flow rate and ACN concentration in mobile phase B, (C) retention time vs. ACN concentration in mobile phase B and flow rate, (D) retention time vs. buffer concentration in mobile phase A and flow rate, (E) retention time vs. buffer and ACN concentrations in mobile phase A, and (F) retention time vs. flow rate and column temperature

**Fig. 3.**
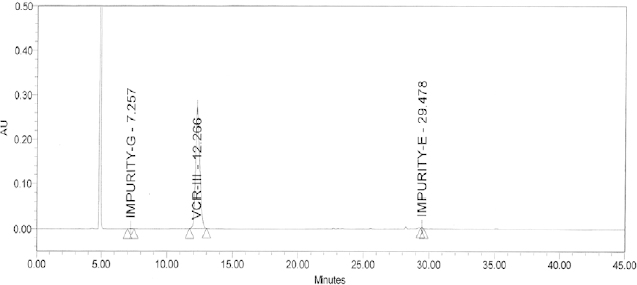
Chromatogram for the optimized method

A validated RP–HPLC method was developed to detect and separate valacyclovir and related impurities (E and G) using the Box-Behnken design approach. The design matrix of the Box-Behnken model of the RSM and experimental runs for the optimization of chromatographic conditions are provided in Table 1. The contour plots of the percent peak area and retention time vs. flow rate, column temperature, acetonitrile (ACN), and buffer in the mobile phase are provided in Figure 2. The details of the linearity data of impurities E and G are provided in Table 3. A representative chromatogram of the optimized method is provided in Figure 3. Results of the method validation showed that the mean recovery (%) of impurity E at the specification level (50, 100, and 150 μg) was 99.9% and the mean recovery of impurity G was 103.2%. For determining the precision of the method, the values of the RSD for the areas of impurities E and G were found to be 0.9% and 0.1%, respectively.

**Fig. 4.**
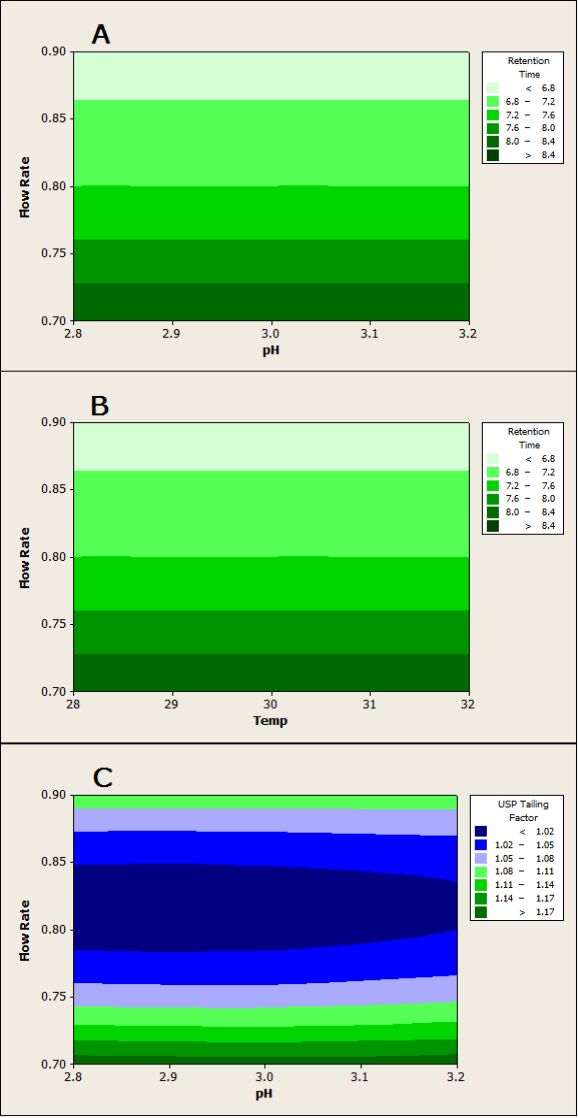
Robustness contour plots of (A) retention time vs. flow rate and pH, (B) retention time vs. flow rate and temperature, and (C) USP tailing factor vs. flow rate and pH

**Fig. 5.**
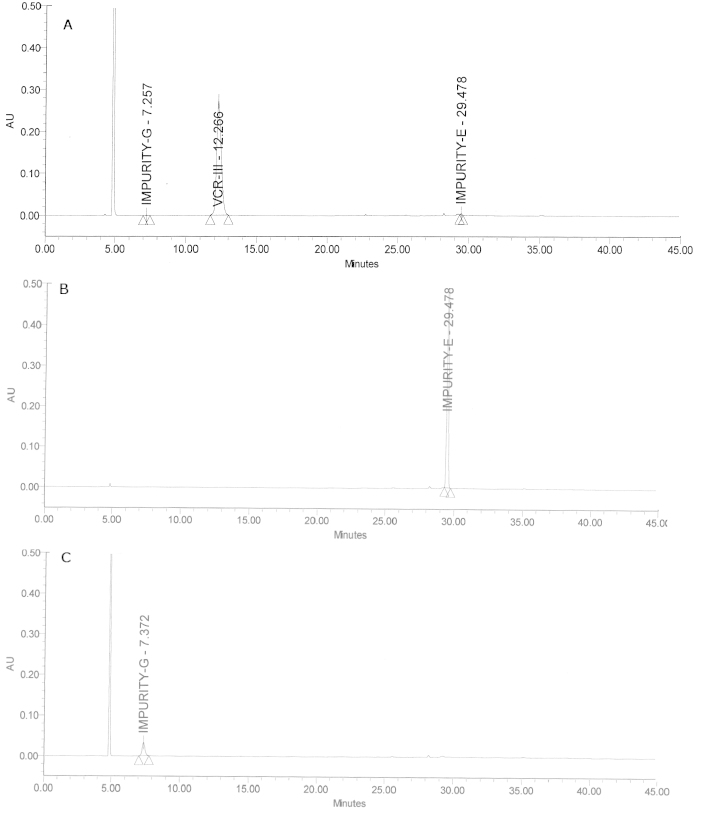
Chromatograms showing (A) ruggedness of the developed method and specificities of (B) impurity E and (C) impurity G

The design matrix of the Box-Behnken model of the RSM and experimental runs for the robustness of the method are provided in Table 2 and Figure 4. Data on the method’s ruggedness are provided in Table 4 and Figure 5. System suitability data are provided in Table 5 showing that it complies with the specified limits. The HPLC chromatogram recorded under the optimized conditions (Figure 3) showed that the retention times of VCR and impurities E and G were 12.2, 29.4, and 7.2 min, respectively. Finally, it was observed that there were no blank interferences with analytes like VCR or impurities E and G (Figure 5), suggesting the specificity of the developed method. The LOD values of the proposed method were found to be 0.0024 μg/mL for impurity E and 0.04 μg/mL for impurity G. The S/N ratios of impurities E and G were calculated as 4.4 and 4.3, respectively. The LOQ values of impurities E and G by the proposed method were found to be 0.0082 μg/mL and 0.136 μg/mL, with S/N ratios of 10.14 and 10.01, respectively. All parameters of method validation lie within the specified limits as per ICH guidelines [4–10].

**Tab. 2.** Design matrix of the BBD for method robustness

Std order	Run order	Pt Type	Blocks	Flow rate (mL/min)	Temp. (°C)	pH	Retention time (min)	USP tailing factor
13	1	0	1	0.8	30	3	7.2	1.01
15	2	0	1	0.8	30	3	7.2	1.01
11	3	2	1	0.8	28	3.2	7.2	1.02
7	4	2	1	0.7	30	3.2	8.4	1.19
5	5	2	1	0.7	30	2.8	8.4	1.19
10	6	2	1	0.8	32	2.8	7.2	1.01
1	7	2	1	0.7	28	3	8.4	1.18
2	8	2	1	0.9	28	3	6.7	1.1
3	9	2	1	0.7	32	3	8.4	1.19
6	10	2	1	0.9	30	2.8	6.7	1.1
9	11	2	1	0.8	28	2.8	7.2	1.01
12	12	2	1	0.8	32	3.2	7.2	1.02
14	13	0	1	0.8	30	3	7.2	1.01
4	14	2	1	0.9	32	3	6.7	1.1
8	15	2	1	0.9	30	3.2	6.7	1.1

**Tab. 3.** Linearity data of impurities E and G

Analyte	Linearity level%	Concentr. (μg/mL)	Peak area	Slope	Y intercept	R^2^ value
Impurity E	I	0.2615	8663	33493	-116.47	1.00
	II	0.4184	13904			
	III	0.523	17369			
	IV	0.6276	20871			
	V	0.7845	26196			
Impurity G	I	0.254	1240	4968.1	-2.3931	0.99
	II	0.4064	2034			
	III	0.508	2520			
	IV	0.6096	3053			
	V	0.762	3760			

**Tab. 4.** Results for ruggedness of impurities

Analyte	USP resolution	USP Plate Count	USP Tailing
Impurity E	42.95	419656	1.23
Impurity G	10.20	6794	1.08

**Tab. 5.** System suitability results for impurities

Analyte	S/N ratio	RSD (%)	USP tailing	USP plate count
Impurity E	38.10	1.1	1.25	439790.97
Impurity G	37.08	0.3	1.17	5948.46

## Experimental

### Drugs and Chemicals

Valacyclovir (VCR) was a kind gift from Torrent Pharmaceuticals, Ahmedabad, India. Impurities E and G were obtained from Sigma Aldrich, Mumbai, India. Potassium dihydrogen phosphate, orthophosphoric acid, acetonitrile, methanol, and water used were either AR grade or HPLC grade, purchased from Merck and Sigma Aldrich, Mumbai, India.

### Instruments

The HPLC system (Shimadzu, LC 10 ADVp, Japan with UV detector) and electronic analytical balance (Shimadzu, Japan) were used.

### Software

Experimental design, data analysis, and contour plots were performed using the Minitab® v16.2.4 trial version.

### Optimization of the Chromatographic Conditions by the Box-Behnken Design Approach

Before proceeding to the optimized RP–HPLC chromatographic conditions for impurity profiling, some preliminary trials were carried out with different gradient mobile phase compositions. Amongst the trials, the UV detector wavelength (254 nm) and injection volume (20 uL) were kept constant. A Box-Behnken design approach of RSM was employed to evaluate the effect of four independent factors, viz., the buffer concentration in mobile phase A in v/v (X1), ACN concentration in mobile phase B in v/v (X2), flow rate (X3) and column temperature (X4) on retention time (Y), and retention ratio (Y1) and percentage peak area (Y2). On the basis of the preliminary experiments, the value ranges of the variables selected in the design were as follows: buffer concentrations in mobile phase A (X1), 90–100 v/v; ACN concentration in mobile phase B (X2), 90–100 v/v, flow rate (X3), 0.8–1.0 mL/min, and column temperature (X4), 25–30°C. The design matrix of the Box-Behnken model of the RSM and experimental runs is provided in Table 1. The equation generated was a polynomial quadratic equation.

Thus, the optimized chromatographic conditions for the separation of impurities include: gradient elution through the Inertsil ODS 3V (250×4.6 mm, 5.0 μm) column using a pH 3 buffer: acetonitrile (95:5, v/v) as mobile phase A and acetonitrile: methanol (90:10, v/v) as mobile phase B and buffer: acetonitrile (50:50, v/v) as diluent. The injection volume was 20 μL with a flow rate of 0.9 mL/min and a total run time of 40 min. The UV detector was set at 254 nm. Experimentation was done at a column temperature of 30°C. Ethanol was used as the needle wash solution and water: acetonitrile (80:20, v/v) was used as a seal wash solution.

### Preparation of the Standard and Stock Solutions

The stock solutions of the impurities were prepared by dissolving 10.45 mg of impurity E and 10.15 mg of impurity G separately in 100-mL volumetric flasks, initially dissolved in 10 mL diluent and the volume was adjusted with the same. From the stock solutions, 0.5 mL each of the solutions of impurities E and G were taken in two separate 100-mL volumetric flasks and diluted up to the mark with diluents to get a concentration of 0.5 μg/mL

The spike solution was prepared by dissolving accurately weighed 10 mg of the VCR sample in a 10-mL volumetric flask, to which 0.5 mL each of the impurity E and G stock solutions were added along with 1 mL of the diluents, well sonicated to ensure complete dissolution of the drug, and volume-adjusted with the diluent.

### System Suitability

To assess the system suitability of the method, the repeatability, theoretical plate count, tailing factor, and retention time of six replicate injections of the standard solutions were used and the percentage relative standard deviation (RSD) values were calculated in each case. Diluent as a blank was injected followed by six replicates of standard preparations and the chromatograms were recorded. The system is said to be suitable only if the signal/noise ratio (S/N) of each impurity in the standard solution is at least 30, the RSD is less than 2%, and the tailing factor does not exceed 2.0 [4–7].

### Validation

The developed RP–HPLC method for the detection and segregation of impurities E and G was validated as per ICH guidelines [4–7]. The linearity of the method for impurities was studied over the concentration range of 50–150 μg/mL and the R^2^ value was not less than 0.99 and the RSD of the peak areas of the solution was not more than 2%, which was regarded as the acceptance criterion. The accuracy of the proposed method was ascertained by recovery studies carried out in triplicate using a standard addition method where the drug substance was spiked with each of the impurities in the concentrations of 50%, 100%, and 150% and the acceptance criteria for the recovery of impurities were set at 90%–110%. The solution used for determining the method precision was prepared with 10 mg VCR, 10 mL of diluents, 0.5 mL of Imp-E and G stock solutions, and volume adjusted to 100 mL with diluents. The method precision was determined by injecting this solution six times into the HPLC system and the % RSD values for the peak areas of Imp-E and Imp-G were calculated. Intermediate precision or ruggedness was carried out by different analysts on different instruments and on different days. The specificity of the method was studied to see if there were any interferences of the blank or excipients at the retention times of the impurities. The blank (diluent) was injected, followed by six replicates of standard preparations; 20 μL of impurities E and G; and spike solutions were injected separately and chromatograms were recorded.

For the measurement of robustness, the BBD approach of RSM was employed to evaluate the effect of three independent factors, viz., flow rate (X1), column temperature (X2), and buffer pH (X3) on responses like retention time (Y) and USP tailing factor (Y1) [8–10]. Basing on previous experimentations and knowledge, the ranges of values used in the design were set as follows: flow rate (X1): 0.7–0.9 mL/min; column temperature (X2): 28–32°C; and buffer pH (X3): 2.8–3.2. The design matrix of the Box-Behnken model of the RSM and experimental runs are provided in Table 2. The equation generated was a polynomial quadratic equation.

The LOD is the measurement of the lowest concentration of impurities that can be detected but not be quantified. The S/N ratio value shall be at least 3 for the LOD solution. The LOQ is the measurement of the lowest concentration of impurities E and G that can be quantified with acceptable precision. The S/N ratio value shall be at least 10 for the LOQ solution. The LOQ stock solution was prepared based upon the S/N ratio obtained by the standard solution. About 0.9 mL of impurity E and 13.6 mL of impurity G stock solutions were taken in 100-mL volumetric flasks, dissolved, and volume-adjusted with diluent. One mL of the above LOQ stock solution was taken into a 100-mL volumetric flask and volume-adjusted with diluent. An amount of 3.3 mL of LOQ solution was taken into a 10-mL volumetric flask, dissolved, and volume-adjusted with diluents to get the LOD stock solution.

## Conclusion

As depicted by ICH guidelines and stated in different pharmacopoeia specifications, impurity profiling is of crucial importance in drug synthesis, quality control, and storage. The developed and optimized RP–HPLC method established by the BBD approach was found to be simple, accurate, robust, precise, and specific. It was also found suitable in terms of tailing factor and theoretical plate count. The two VCR process-related impurities were well-separated with good resolution and peak shape, good retention times, and hence this method could be applied in impurity profiling of valacyclovir drug-related substances.
